# The multiple uses of telemedicine during the pandemic: the evidence from a cross-sectional survey of medical doctors in Brazil

**DOI:** 10.1186/s12992-022-00875-9

**Published:** 2022-09-19

**Authors:** Mário Scheffer, Alex Cassenote, Maria Teresa Seabra Soares de Britto e Alves, Giuliano Russo

**Affiliations:** 1 Department of Preventive Medicine, University of São, Paulo. Avenida Dr. Arnaldo, 455, 2º andar, sala 2166, São Paulo (, SP CEP: 01246-903 Brazil; 2 Program in Public Health, Federal University of Maranhão, Rua Barão de Itapary, Nº 155, Centro, São Luís, MA CEP: 65020-070 Brazil; 3 Wolfson Institute of Population Health, Queen Mary University of London, 58 Turner street, London, E1 2AB UK

**Keywords:** Telemedicine, Telehealth, Health systems, Medical services, Health in low- and middle-income countries, Doctors and physicians

## Abstract

**Background:**

The use of telemedicine, or the provision of healthcare and communication services through distance-based technologies, has increased substantially since the 2019 novel coronavirus (COVID-19) pandemic. However, it is still unclear what are the innovative features of the widespread use of such modality, its forms of employment and the context in which it is used across pluralist health systems, particularly in low- and middle-income settings. We have sought to provide empirical evidence on the above issues by analysing the responses of medical doctors in a representative cross-sectional survey in two states in Brazil: São Paulo and Maranhão.

**Methods:**

We analysed the responses of 1,183 physicians to a survey on the impact of COVID-19 on their livelihood and working practice. Two independent samples per state were calculated based on a total of 152,511 active medical registries in São Paulo and Maranhão. Proportional stratified sampling was performed and the distributions for gender, age, state and location of address (capital or countryside) were preserved. The survey contained questions on the frequency of physicians’ employment of telemedicine services; the specific activities where these were employed, and; the forms in which the pandemic had influenced the adoption or consolidation of this technology. We performed descriptive and univariate analysis based on the chi-square test or Fisher's exact test for the qualitative data, and the Mann–Whitney test in the quantitative cases. Data were shown as absolute frequency and proportion with a 95% confidence interval.

**Results:**

In our sample of physicians, telemedicine was employed as a form of clinical collaboration by most doctors (76.0%, 95 CI 73.6–78.5), but only less than a third of them (30.6%, 95 CI 28.0–33.3) used it as a modality to provide healthcare services. During the pandemic, telemedicine was used predominantly in COVID-19-related areas, particularly for hospital-based in-patient services, and in private clinics and ambulatory settings. Male, younger doctors used it the most. Doctors in São Paulo employed telemedicine more frequently than in Maranhão (*p* < 0.001), in urban settings more than in rural areas (*p* < 0.001). Approximately three-quarters of doctors in large hospitals reported using telemedicine services (78.3%, 95 CI 75.9–80.6), followed by doctors working for smaller private clinics (66.4%, 95 CI 63.7–69.1), and by a smaller proportion of primary care doctors (58.4%, 95 CI 55.6–61.2).

**Conclusions:**

Our study suggests that telemedicine may have helped ensure and expand the range of communication and healthcare services in low- and middle-income settings during the COVID-19 pandemic. However, the modality appears to lend itself to be disproportionally used by doctors working in specific, priviledged sections of pluralistic health systems, and presumably by patients seeking care there. Regulation and incentives will be required to support the use of the technology across health systems in low- and middle-income countries in order to increase access to services for less disadvantaged populations.

**Supplementary Information:**

The online version contains supplementary material available at 10.1186/s12992-022-00875-9.

## Background

Telemedicine or telehealth may be defined as the remote provision of health services through the use of communication and information technologies, such as telephone, videoconferencing, email or cellphone applications. It encompasses consultations, procedures, storage and transmission of data and health information by means of sound, text or images, aiming at the prevention, diagnosis and treatment of patients, as well as at interaction and training among practitioners and healthcare teams [1, 2].

As a widely used procedure during the COVID-19 pandemic around the world, several authors have reported that telemedicine helped maintain physical isolation and avoid direct physical contact, minimizing the risk of COVID-19 transmission [3]. Teleconsultations were used in triage, the evaluation of suspected cases and as support for the diagnosis and treatment of COVID-19 [4], including in cases of patients admitted to intensive care, as well as enabling access to expert opinions not readily available in services [5].

In primary care services, it has been used successfully in screening suspected cases, referrals to testing and personalized care for mild cases [6]. In other areas not related to COVID-19 treatment, the technology has reduced harm from clinical service closures and elective care cancellations. It has been used in the management of unavoidable surgeries [7]; the identification of patients in remote and rural areas who required removal and hospitalization [8]; and the management of chronic diseases, such as asthma and immunodeficiencies [9].

Several innovations that have been introduced may be continued post-pandemic, such as the treatment of drug users [10] and of patients with metabolic disorders [11], and the health promotion of young adults [12]. Telemedicine has therefore been incorporated into the continuing education of physicians, the updating of clinical protocols and undergraduate and residency education [13].

The limits to telemedicine use have been studied during the pandemic, including socioeconomic and racial/ethnic barriers that hinder access to this resource [14]; dilemmas about quality of care; the absence of physical exams and imaging; and ethical issues about the doctor–patient relationship, access, consent, and privacy [15].

From the perspective of physicians’ labour, there are obstacles in validating workload and determining compensation amounts for professionals [16], with risks that telemedicine will become a new form of public–private dual-practice for physicians [17], adding to concerns about this type of work that have already been observed in low- and middle-income countries (LMICs) [18].

Although COVID-19 represents a “natural experiment” case for telemedicine, there are gaps in knowledge about its use in the pandemic that, if filled, may contribute to improve the future utilization of this technology by health systems. Such knowledge may also inform the definition of descriptors intended to guide future research on the topic. Among the unanswered questions in the literature that are the subject of our study, it is still unclear how telemedicine has been used by different components of the same health system, what the main purposes of its use are, and which professionals and types of service have most frequently benefited from this resource.

These questions are particularly relevant for LMICs, where health systems and services are heterogeneous, where there are different forms of financing and where the combination of public and private sectors is complex [19].

The study defined and tested four hypotheses based on the literature: 1) A large percentage of physicians had never used telemedicine, but did so during the pandemic; 2) telemedicine was used as a tool to mitigate the closure of services and the postponement of elective treatments, and was used more frequently by physicians who did not provide COVID-19 services than by physicians providing COVID-19 services; 3) telemedicine use was more intense in primary care/ambulatory care than in hospital care; and 4) telemedicine use was higher by physicians in the private and mixed sectors and lower among physicians working exclusively in the public sector, based also on studies that have demonstrated inequalities in access to telemedicine determined by service supply and social conditions.

The basic research for this article was conducted in Brazil, a country with the second highest burden of COVID-19 in the world, where the course of the pandemic was marked by socioeconomic inequalities [20], regional inequalities [21] and diversity of funding, infrastructure, and workforce allocation in sub-national health systems [22]. To encompass profiles of telemedicine use in diverse conditions affecting services, human resources and population health demands, we chose to study two extremely different Brazilian states: São Paulo and Maranhão.

Located in the northeast region of the country, Maranhão has a lower human development index and lower level of health funding compared to São Paulo [23], a larger dependence on the public network, and a lower offer of physicians per thousand inhabitants [24]. São Paulo, in turn, was more strongly affected by the pandemic, registering worse indicators of morbidity and mortality by COVID-19.

Telemedicine was used during the pandemic in Brazil in order to assess the severity of the waves of COVID-19, considering emergency room referral as a marker of more severe disease evolution [25]. Updated treatment protocols and telehealth training were offered to frontline professionals, physicians, nurses and physical therapists [26]. Teleconsultation was implemented to monitor patients vulnerable to infection (such as those with mental, respiratory and nutritional disorders) [27], for patients in paediatric programmes [28], for post-COVID-19 respiratory physiotherapy [29] and for remote care for dementia patients, with good recruitment and compliance rates [30].

A relevant share of physicians performed teleconsultations during the pandemic, but there was more frequent care for patients in the private sector than for patients in the public sector [31]. COVID-19 accelerated the regulation of telemedicine practice in Brazil [32] and the digitalization of private health insurance companies, but there are criticisms about the poor remuneration of physicians and the expanded use of telemedicine to reduce care costs in the private sector, even if worsening quality of care for patients has also been mentioned.

Although telemedicine has been the subject of a wide range of studies, there is still scarce empirical evidence on the different uses of this technology during the pandemic in LMICs, in different contexts and by different actors. Our study sought to make a contribution to this body of literature from the experience of physicians in two states at different stages of development in Brazil.

## Methods

This survey is part of a wider study on the impact of COVID-19 and economic recessions on Brazil’s health system and workforce conducted in 2021. In the survey, physicians were asked to report information on their COVID-19 and vaccination status, on the changes of workloads and earnings experienced during the pandemic, and on the impact on working practices such as telemedicine. This paper focuses on the responses on the latter topic.

### Data collection

The physicians’ database for the two states was provided by Brazil’s Federal Council of Medicine; the survey sample was calculated by the School of Medicine of the University of São Paulo; and the actual survey was carried out by the survey services institute “Datafolha”, under the technical supervision of the academic partners of the study.

A representative cross-sectional study was performed and included a sample of 1,183 physicians. This sample size was calculated from the total population size of 152, 511, which was generated from medical registries of Sao Paulo which had a total of 144,852, and Maranhao with 7, 659 physicians. Proportional stratified sampling was performed and the distributions for gender, age, state and location of address (capital or countryside) were preserved. Substitutions were made in cases of unsuccessful contact or refusal to participate in our survey; 1,183 physicians were randomly selected, and five substitutions were identified for each sampled physician.

Primary data were collected via telephone survey carried out by eight data collectors, including one field coordinator, six experienced interviewers and two administrative staff responsible for checking missing data.

### Variables and statistical analysis

The survey questionnaire was designed specifically for this research project and aimed at identifying the socioeconomic profile of the selected physicians, the type and specificities of the workplaces and the workload and type of contractual relationship between physician and employer, particularly during the period of the COVID-19 pandemic (see the complete survey questionnaire in Additional file 1). The specific telemedicine questions and respective variables aimed to ascertain the type of clinical and non-clinical functions carried out through distant technology; whether the use of this modality predated the pandemic or had only been introduced recently; and the number of hours dedicated to the use of this modality over an average week (Table 1).

**Table 1:** Telemedicine questions and variables in the survey

Specific survey question	Answer/variable
Q.21. Do you currently carry out any of the following professional activities: a)Carry out appointments and guidance to patients by telemedicine? b)Have work meetings by telemedicine? c)Have case discussions with colleagues by telemedicine? d)Perform prescriptions, certificates or reports by remote methods or telemedicine? e)Prepare/annotate electronic medical records by telemedicine? f)Receive medical qualification or training by telemedicine?	YES (1); NO (2)
Q.21a Do you perform any other activity by telemedicine? Which ones?	(WRITE DOWN THE SPECIFIC ACTIVITY)
Q.22 (IF Q.21 = 1 IN AT LEAST OE ITEM) Considering your relationship with telemedicine/remote consultation, you: 1.had already been using this resource before the pandemic and kept using it 2.had never used this resource, but started using it due to the pandemic OR 3.used the resource before, but are no longer using it since the beginning of the pandemic 4.had already used telemedicine occasionally to receive training, but never used it with patients (SPONTANEOUS ANSWER)	YES (1); NO (2)
Q.23 (IF Q.22 = 1 or 2) Currently, in your professional activities, how many hours (on a weekly basis) do you devote to digital platforms/telemedicine/remote consultation?	WRITE DOWN THE SPECIFIC NUMBER OF HOURS

Source: USP-UFMA-QMUL (2021): Physicians’ survey regarding work and impact of COVID-19

The main variables from the survey used for stratification were (1) administrative type of the physicians’ employer (public, private and dual practice in the case of overlapping employment relationships); and (2) type of work in the healthcare facilities (public, primary and outpatient care; private individual offices and clinics; public or private hospitals and non-assistance administrative services) (see the complete survey questionnaire in  Additional file 1).

We performed descriptive and univariate analysis based on the chi-square test or Fisher's exact test for the qualitative data and the Mann–Whitney test in the quantitative case to understand the use of telemedicine among these physicians. Data were shown as absolute frequency and proportion with a 95% confidence interval. The database developed by the Datafolha data collectors was exported to the Statistical Package for the Social Sciences (SPSS) version 26 for Windows (International Business Machines Corp, New York, USA) and R-GUI version 3.5.3 for statistical treatment. All the significance levels were set to *p* < 0.05.

## Results

In the first part of this section, we show the evidence from our survey on the frequency, time spent and purposes of telemedicine use by medical professionals during the COVID-19 pandemic. Thereafter, we present data on the profile of the medical providers of telemedicine services in the two states, as well as on the types of service where these professionals carry out their practice. Finally, we address the telemedicine used by physicians in COVID-19 intervention centres and in other types of care.

### Purposes of telemedicine use during the pandemic

**Fig. 1:**
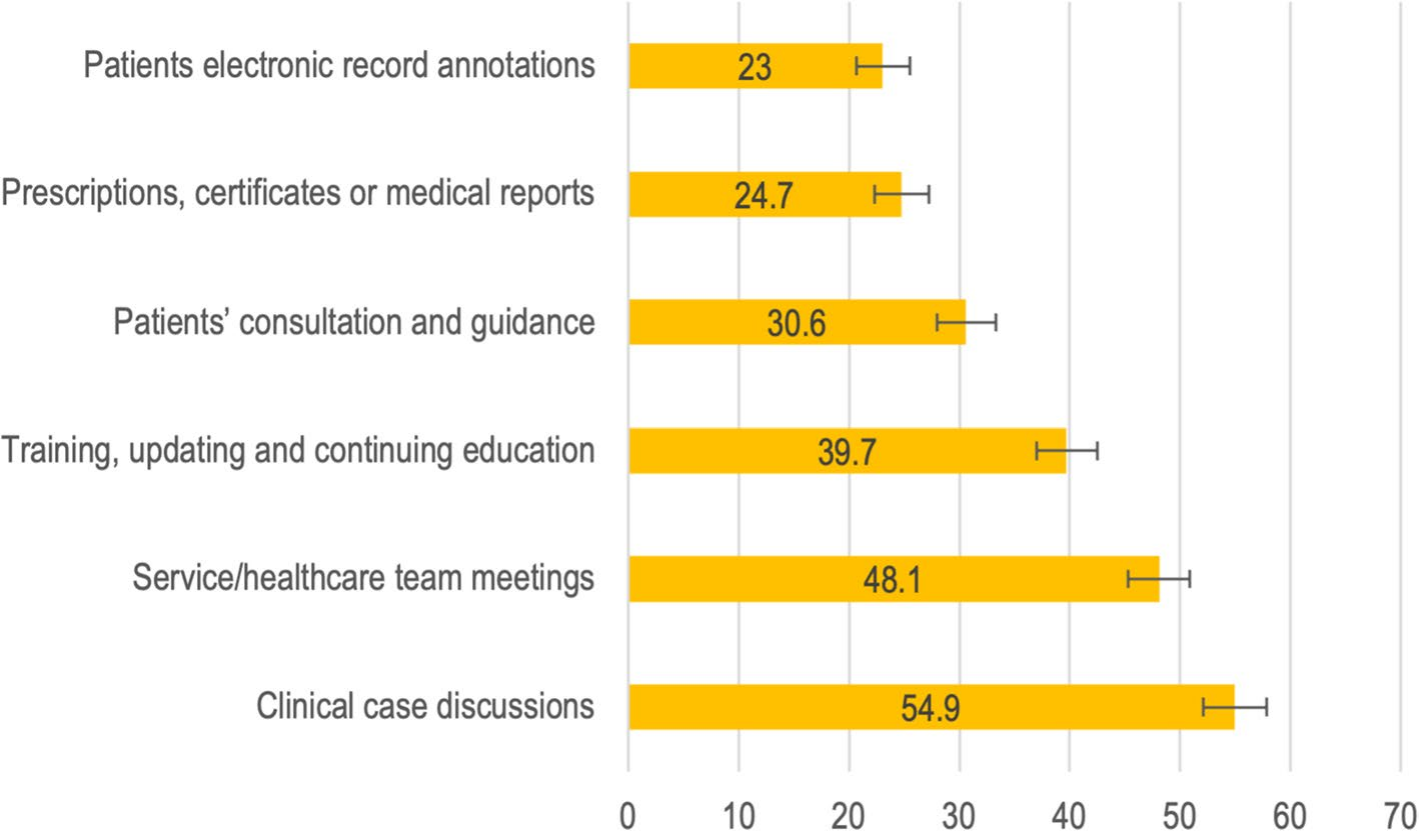
Purpose of telemedicine use by physicians during the COVID-19 pandemic – proportion of respondents. Source: University of São Paulo (USP- Federal University of Maranhão (UFMA)- Queen Mary University of London (QMUL) (2021): Physicians’ survey regarding work and impact of COVID-19

Most physicians (76.0%, 95 CI 73.6–78.5) reported using telemedicine resources, with a significant difference between physicians working exclusively in the public sector (24.4%, 95 CI 21–4-27.8) and those working in dual public–private practice (62.3%, 95 CI 59.1–65.5) (Fig. 1 and Table A1 in  Additional file 2).

On average, physicians reported devoting between six and eight hours per week to telemedicine services, with significant differences between public and private sector physicians (*p* = 0.031); between younger and older physicians (*p* = 0.059); and between physicians working in the countryside or in the capital city health facilities (*p* = 0.076) (see Table A2 in Additional file 2).

Telemedicine was mostly used to connect professionals in discussing clinical cases (54.9%, 95 CI 52.1–57.8), for in-service meetings (48.1%, 95 CI 45.3–50.9) and in training and knowledge updates (39.7%, 37.0–42.5). Less than a third of physicians stated that they performed consultations and guided patients (30.6%, 95 CI 28.0–33.3), a practice more commonly known as “teleconsultation” (see Table A3 in Additional file 2).

### Characteristics of physicians who use telemedicine

Table 2 below shows the characteristics of physicians according to the purpose for which they use telemedicine. Significant differences were found in all forms of use among physicians who work exclusively in the public sector or exclusively in the private sector.

**Table 2:** Profile of physician users of telemedicine and purposes of its employment

	Office visits and guidance			Healthcare team meetings			Case discussions			Prescriptions and certificates			Patient record annotations			Training		
	n		*p*-value	n		*p*-value	n		*p*-value	n		*p*-value	n		*p*-value	n		*p*-value
Sex																		
Male	190	52.5	0.086	303	53.3	0.053	352	54.2	0.115	150	51.4	0.055	143	52.6	0.183	259	55.1	0.533
Female	172	47.5		266	46.7		298	45.8		142	48.6		129	47.4		211	44.9	
Age																		
< 35	112	30.9	0.250	187	32.9	0.079	230	35.4	0.024	86	29.5	0.078	79	29.0	0.025	147	31.3	0.1600
35–50	134	37.0		213	37.4		236	36.3		114	39.0		111	40.8		174	37.0	
> 50	116	32.0		169	29.7		184	28.3		92	31.5		82	30.1		149	31.7	
Sector																		
Private	70	19.3	< 0.001	81	14.2	0.032	75	11.5	0.112	49	16.8	0.001	56	20.6	< 0.001	71	15.1	0.158
Public	71	19.6		126	22.1		158	24.3		53	18.2		44	16.2		112	23.8	
Dual-practice	221	61.0		362	63.6		417	64.2		190	65.1		172	63.2		287	61.1	
State																		
Maranhão	153	42.3	0.048	219	38.5	< 0.001	283	43.5	0.022	127	43.5	0.225	98	36.0	< 0.001	218	46.4	0.953
São Paulo	209	57.7		350	61.5		367	56.5		165	56.5		174	64.0		252	53.6	
Region																		
Countryside	164	45.3	0.020	258	45.3	< 0.001	316	48.6	0.144	117	40.1	< 0.001	125	46.0	0.084	229	48.7	0.313
Capital	198	54.7		311	54.7		334	51.4		175	59.9		147	54.0		241	51.3	

Source: USP-UFMA-QMUL (2021): Physicians’ survey regarding work and impact of COVID-19

Dual-practice physicians used telemedicine more for teleconsultations (*p* < 0.001), drug prescriptions and certificates (*p* = 0.001) and electronic medical records (*p* < 0.001).

Physicians in São Paulo state reported more frequent use of telemedicine than physicians in Maranhão, particularly for in-service meetings and medical records (both *p* < 0.001) and for case discussion (*p* = 0.002).

Physicians based in areas of the capital used telemedicine significantly more than physicians in the countryside, especially for meetings and prescriptions (both *p* < 0.001) and for teleconsultations (*p* = 0.020).

Younger (< 50 years) and male physicians showed a higher tendency to use telemedicine services compared to older and female physicians, but with lower levels of significance.

### Types of service where physicians practice telemedicine

**Fig. 2:**
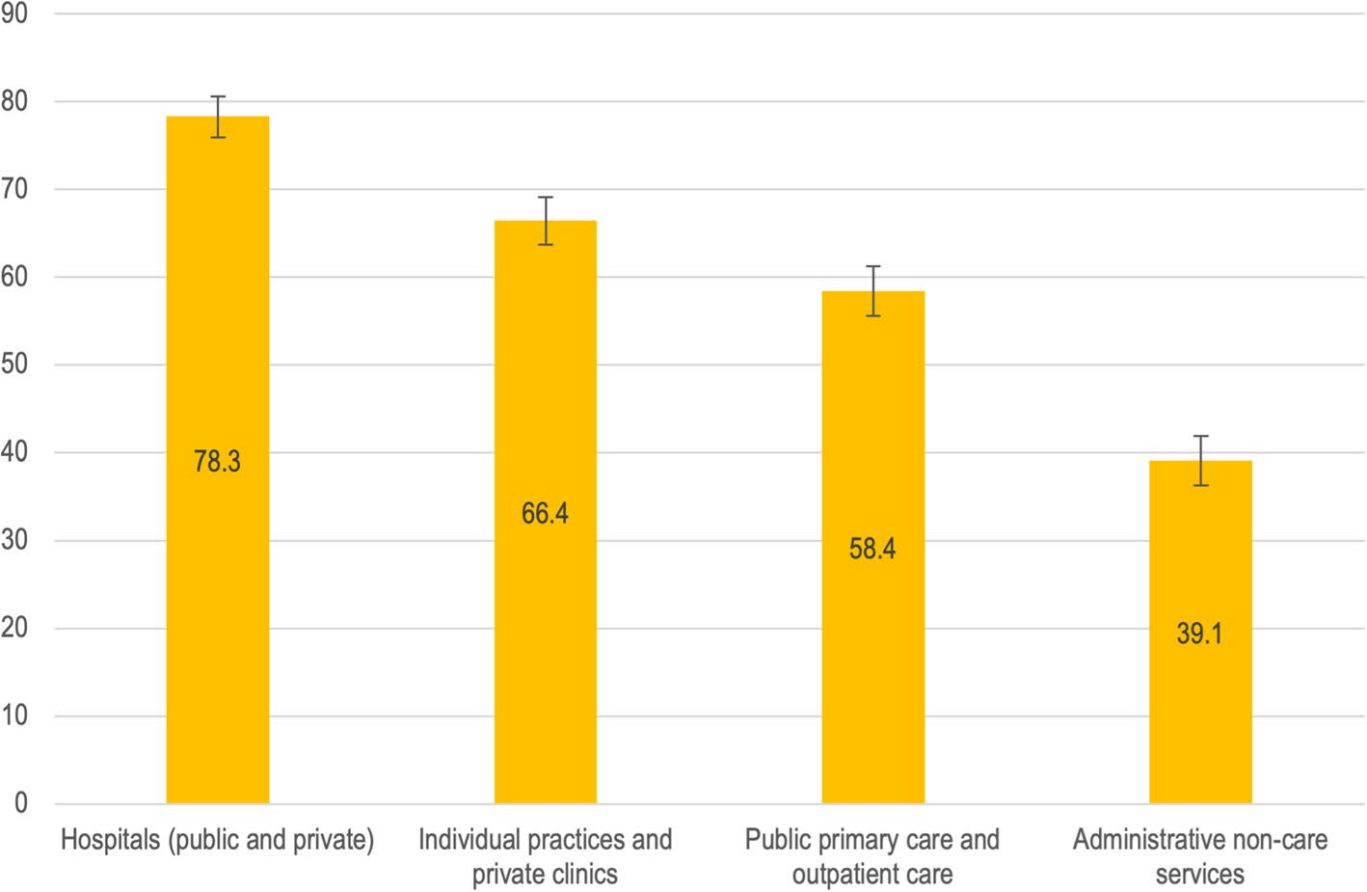
Types of service provided by physicians as users of telemedicine. Source: USP-UFMA-QMUL (2021): Physicians’ survey regarding work and impact of COVID-19

According to the type of service (Fig. 2 and Table A4 in Additional file 2), most physicians who work in large public and private hospitals stated that they used telemedicine (78.3%, 95 CI 75.9–80.6), followed by physicians working in private non-hospital services (isolated offices and private clinics) (66.4%, 95 CI 63.7–69.1). On the other hand, 58.4% of physicians working in public non-hospital services (basic units, primary care and specialized outpatient clinics) stated that they had used telemedicine; (95 CI 55.6–61.2).

### Use of telemedicine by type of healthcare service

Among physicians working in outpatient services in the public sector (including primary care), telemedicine was mostly used for COVID-19-related services (63.7%, 95 CI 63.2–70.0). But among physicians working in private practices and clinics, telemedicine for non-COVID-19-related services prevailed (78.1%, 95 CI 74.0–81.8) (Table 3).

**Table 3:** Use of telemedicine by physicians who directly cared for COVID-19 cases and physicians who did not work with COVID-19, according to type of health service

Type of services	Not worked with COVID-19				Worked with COVID-19				
	n	%	CI inf	CI sup	n	%	CI inf	CI sup	*p*-value
Public primary care and outpatient care									
No	242	55.90	51.20	60.50	250	33.30	30.00	36.80	< 0,001
Yes	191	44.10	39.50	48.80	500	66.70	63.20	70.00	
Individual practices and private clinics									
No	95	21.90	18.20	26.00	302	40.30	36.80	43.80	< 0,001
Yes	338	78.10	74.00	81.80	448	59.70	56.20	63.20	
Hospitals (public and private)									
No	123	28.40	24.30	32.80	134	17.90	15.30	20.70	< 0,001
Yes	310	71.60	67.20	75.70	616	82.10	79.30	84.70	
Administrative non-care services									
No	260	60.00	55.40	64.60	461	61.50	57.90	64.90	0,665
Yes	173	40.00	35.40	44.60	289	38.50	35.10	42.10	

Source: USP-UFMA-QMUL (2021): Physicians’ survey regarding work and impact of COVID-19

Among physicians working in hospitals (in-patient care) in both the public and private sectors, telemedicine was mainly employed in in-patient services for patients with COVID-19 (82.1%, 95 CI 79.3–84.7).

Teleconsultation for outpatient care and patient guidance was prevalently used in non-COVID-19 activities, that is, for regular medical care (74.1%, 95 CI 70.9–77.2).

## Discussion

In our sample from Brazil, telemedicine was employed by most physicians and was directed towards professional interactive activities and the discussion of clinical cases. Only a third of physicians used it for teleconsultation and patient guidance services. During the pandemic, telemedicine was used by physicians working in COVID-19-related services, mainly in hospitals, offices, outpatient clinics and private clinics. Male, younger, dual-practicing physicians working in areas of the capital and in São Paulo were the largest users of this technology. More than three-quarters of physicians in public and private hospitals reported using telemedicine, followed by physicians in private offices and clinics. Only a minority of primary care physicians reported using telemedicine.

These results have to be considered taking into account some limitations. First, the study survey aimed to measure the overall impact of COVID-19 on medical work in Brazil, and some questions were related to the use of telemedicine during the pandemic. We therefore did not go into depth on the distinction between the various uses of telemedicine [33] in each type of service, or the implications of the different technological platforms available [34]. Second, the complexity of the organization and financing of the Brazilian health system did not allow us easily to identify where physicians practice, as many have multiple jobs and concomitant public and private practice [35]. We chose, based on the individual doctors' main places of practice, to divide them between public, private and dual-practice; and between hospital, public primary care/ambulatory, private outpatient and non-assistance services. Finally, the states of São Paulo and Maranhão have very specific development and health system characteristics, which should be taken into account in the eventual comparison with other low- and middle-income states or LMICs [23]. However, we consider our findings and reflections valid and pertinent to other contexts.

The study showed that most physicians in our sample adopted telemedicine to perform a multiplicity of functions, such as clinical collaboration, healthcare team meetings, professional updating and patient care. Only a minority of physicians stated that they performed consultations and guided patients—a practice more commonly known as “teleconsultation”. While this multipurpose application of telemedicine confirms findings from other studies [3], the results also indicate that the pandemic may have expanded the frequency of telemedicine employment modalities beyond teleconsultation.

Prior to COVID-19, most reviews regarding telemedicine focused on effectiveness, cost-effectiveness, implementation and patient experience [36]. In the future, reviews will probably need to distinguish between the different forms and functions of telemedicine, including a better delimitation of the concepts of telehealth, telemedicine and teleconsultation, which today are usually interchangeable in the literature.

Our study emphasizes how the main use of telemedicine occurred in the shared discussion of clinical cases, both in the care of COVID-19 and to replace services that had been interrupted due to the pandemic. It therefore proved to be a particularly useful tool in cases where there is a need to obtain in-depth knowledge for diagnostic decisions or patient treatment, which require multidisciplinary knowledge or knowledge from more than one medical speciality.

In post-COVID-19 contexts, the use of telemedicine to discuss cases and healthcare team meetings (another use identified in the study) may be useful in several situations: in critical events and in routine situations, both in primary care and in hospitals; in in-service care and teaching; in remote and rural areas with a shortage of professionals; and in services without the presence of certain medical specialists, or even in the shared management of services and in the management of complex health problems that require a multidisciplinary team [37].

Another frequent use of telemedicine that was identified in the study as promising is in terms of training, knowledge-updating and continuing education. Distance education activities (through courses, classes, lectures, discussion and doubt-clearing forums) are capable of quickly updating professionals regarding emerging health problems and guidelines that are in constant evolution. But telemedicine can also constitute an alternative for the present programmes of on-the-job training of human resources to keep them updated on rapidly evolving health programmes and policies, medical specialities, clinical guidelines and therapeutic consensus. The expectation is that, in the future, telemedicine will allow the maintenance of quality medical training, even during possible health emergencies [38].

One-third of the physicians in our sample reported having performed teleconsultations at a higher frequency than during pre-pandemic periods. COVID-19 accelerated the regulation of this practice in several countries, and this will require monitoring its eventual expansion. There will be limits to the growth of teleconsultation [36] because, in certain specialities and for certain health problems, telemedicine is definitely not the most effective form of care. Despite its lower cost and reasonable acceptance among practitioners, some of the barriers to the more expanded use of telemedicine include the low quality of non-face-to-face care; problems with patients’ medical records and notifiable disease reporting; physicians’ compensation; and ethical issues involving the physician–patient relationship and data sharing.

Our data seem to confirm that it is mainly young, private sector physicians from urban areas who have most frequently adopted telemedicine, and that it has mostly been used in São Paulo, the more developed Brazilian state. The more frequent use of telemedicine in urban areas has been identified already in other studies [39], but other aspects will deserve attention from future research; in segmented health systems in LMICs where private spending predominates over public (as is the case in Brazil), the expansion of telemedicine may be commercially exploited by popular clinics and health plans sold at lower prices, increasing out-of-pocket costs (albeit such forms of care lack the ability to follow up cases until the resolution and lack linkages to other services in the system).

On the other hand, telemedicine may worsen existing inequalities of access to services. As has already been pointed out by other studies [14], digital inequalities in the diffusion and adoption of new technologies mean the most socially vulnerable patients with greater health needs use telemedicine the least, a phenomenon described in literature as the “Inverse Care Law” [40].

In the context of caring for COVID-19, telemedicine was used more by physicians in hospitals (public and private) and in private practices and outpatient clinics, and only by a minority of primary care physicians. In Brazil, as in other LMICs, there will be a need to improve the legislation and regulation of the use of telemedicine [41], as well as a need to review human, structural, political and institutional capabilities for the better use of this technology [42].

Nationwide public policies for the use of telemedicine should consider the multipurpose potential of the technology for patient care, integration of services, sharing of expertise and the continuing education of professionals. The technologies needed for telemedicine (internet, computers, etc.) and the training of professionals to match the needs of patients should reach public services and municipalities far from capital cities.

In the specific case of LMICs, the COVID-19 pandemic has demonstrated that telemedicine can be an additional and useful tool in services that would be absent if this technology did not exist. Despite its proven limitations, it holds out the prospect of being employed beyond its original objectives, offering new opportunities to support health systems and human capabilities.

## Conclusions

It has been documented that during the COVID-19 pandemic, the use of remote telehealth services increased dramatically in the health sector as an instrument to guarantee essential communications and as a way to provide basic services to remote patients. Little empirical evidence exists, however, on the modalities of employment of such technology in LMICs, or on its impact on maintaining provision of care. This matters, as in most of such countries scarce health professionals cannot be deployed to cover remote areas, and technology might be used creatively to mitigate gaps. We used data from a cross-sectional survey of medical doctors from two states in Brazil to explore the uses, modalities and effects of telehealth services in the different levels of the country’s complex health sector.

Our analysis showed that the pandemic has increased both the frequency and range of the modalities of this technology across the different parts of Brazil’s health system, and that communication and collaboration activities across health professionals-rather than distance-based visits-were the most frequent reasons for its use. Male, younger doctors working directly with COVID-19 services were the most frequent users of this technology, particularly those working in private or mixed hospital settings in São Paulo state.

The present study suggests that telemedicine has in unsuspected ways to guarantee and expand the provision of health services in low- and middle-income settings during the COVID-19 pandemic. However, it appears that the modality lends itself to be used more frequently by medical doctors in specific parts of the health system. In order to allow telemedicine to be more widely accessible to the most vulnerable sections of the population, it will be key to regulate this modality in Brazil, as well as in other LMICs. Costs, benefits and barriers for expanding services will need to be monitored and reviewed, particularly taking into consideration the medical, social and policy changes prompted by the COVID-19 pandemic.

## Supplementary Information

**Additional file 1.** 

**Additional file 2: Table A1. **Use of telemedicine for specificfunctions and services. **Table A2.** Number of hours dedicated per weekto telemedicine, according to physicians' characteristics. **Table A3.** Purpose of telemedicine use byphysicians during the COVID-19 pandemic. **Table A4.** Types of service provided byphysicians as users of telemedicine. 

## Data Availability

The datasets used and/or analysed during the current study are available from the corresponding author on reasonable request.

## Abbreviations

95 CI: Confidence interval at 95% (Ethics Committee)
CEP: Comité Ético da Pesquisa
COVID-19: 2019 Novel Coronavirus
FAPESP: Fundação de Amparo à Pesquisa de São Paulo (Brazil)
FMUSP: Faculdade de Medicina da Universidade de São Paulo (Brazil)
LMICs: Low- and Middle-Income Countries
MRC: Medical Research Council (UK)
QMUL: Queen Mary University of London (UK)
UFMA: Universidade Federal do Maranhão (Brazil)
USP: Universidade de São Paulo (Brazil)
