# Supplementary material for: The multiple uses of telemedicine during the pandemic: the evidence from a cross-sectional survey of medical doctors in Brazil
Source: Global Health. 2022 Sep 19;18:81. doi: 10.1186/s12992-022-00875-9 (PMC9483882; doi:10.1186/s12992-022-00875-9)
Supplement: Supplementary file 2 — Additional file 2: Table A1. Use of telemedicine for specificfunctions and services. Table A2. Number of hours dedicated per weekto telemedicine, according to physicians' characteristics. Table A3. Purpose of telemedicine use byphysicians during the COVID-19 pandemic. Table A4. Types of service provided byphysicians as users of telemedicine. [file 12992_2022_875_MOESM2_ESM.docx]

# Statistical annex

**Table A1: Use of telemedicine for specific functions and services**

| Purpose of the use of telemedicine | Not performed work with COVID-19 | | | | Worked related to COVID-19 | | | |  |
| --- | --- | --- | --- | --- | --- | --- | --- | --- | --- |
|  | n | % | CI inf | CI sup | n | % | CI inf | CI sup | p-value |
| Office visits and guidance |  |  |  |  |  |  |  |  |  |
| Yes | 168 | 38.80 | 34.30 | 43.50 | 194 | 25.90 | 22.80 | 29.10 | < 0,001 |
| No | 265 | 61.20 | 56.50 | 65.70 | 556 | 74.10 | 70.90 | 77.20 |  |
| Healthcare team meetings |  |  |  |  |  |  |  |  |  |
| Yes | 207 | 47.80 | 43.10 | 52.50 | 362 | 48.30 | 44.70 | 51.80 | 0,904 |
| No | 226 | 52.20 | 47.50 | 56.90 | 388 | 51.70 | 48.20 | 55.30 |  |
| Case discussions |  |  |  |  |  |  |  |  |  |
| Yes | 234 | 54.00 | 49.30 | 58.70 | 416 | 55.50 | 51.90 | 59.00 | 0,671 |
| No | 199 | 46.00 | 41.30 | 50.70 | 334 | 44.50 | 41.00 | 48.10 |  |
| Prescriptions, certificates and medical reports |  |  |  |  |  |  |  |  |  |
| Yes | 129 | 29.80 | 25.60 | 34.20 | 163 | 21.70 | 18.90 | 24.80 | 0,002 |
| No | 304 | 70.20 | 65.80 | 74.40 | 587 | 78.30 | 75.20 | 81.10 |  |
| Patient electronic record annotations |  |  |  |  |  |  |  |  |  |
| Yes | 127 | 29.30 | 25.20 | 33.70 | 145 | 19.30 | 16.60 | 22.30 | < 0,001 |
| No | 306 | 70.70 | 66.30 | 74.80 | 605 | 80.70 | 77.70 | 83.40 |  |
| Training, updating, continuing education |  |  |  |  |  |  |  |  |  |
| Yes | 176 | 40.60 | 36.10 | 45.30 | 294 | 39.20 | 35.80 | 42.70 | 0.666 |
| No | 257 | 59.40 | 54.70 | 63.90 | 456 | 60.80 | 57.30 | 64.20 |  |

**Table A2: Number of hours dedicated per week to telemedicine, according to physicians' characteristics**

| Physicians' characteristics | Average | Std deviation | Median | Percentile 25 | Percentile 75 | p-value |
| --- | --- | --- | --- | --- | --- | --- |
| Sex |  |  |  |  |  |  |
| Male | 6 | 9 | 4 | 2 | 6 | 0.091 |
| Female | 7 | 10 | 4 | 2 | 8 |  |
| Age |  |  |  |  |  |  |
| < 35 | 7 | 10 | 4 | 2 | 8 | 0.059 |
| 35–50 | 6 | 9 | 4 | 2 | 6 |  |
| > 50 | 7 | 9 | 4 | 2 | 10 |  |
| Professional working area |  |  |  |  |  |  |
| Private | 8 | 8 | 5 | 3 | 10 | 0.031 |
| Public | 7 | 7 | 4 | 2 | 8 |  |
| Dual-practice | 7 | 10 | 4 | 2 | 6 |  |
| State |  |  |  |  |  |  |
| Maranhão | 7 | 10 | 4 | 2 | 8 | 0.997 |
| São Paulo | 7 | 9 | 4 | 2 | 8 |  |
| Region |  |  |  |  |  |  |
| Countryside | 6 | 10 | 4 | 2 | 6 | 0.076 |
| Capital | 7 | 9 | 4 | 2 | 8 |  |

**Table A3: Purpose of telemedicine use by physicians during the COVID-19 pandemic**

| **Purpose of the use of telemedicine** | **n** | **CI(95)-lower and upper limit** | | |
| --- | --- | --- | --- | --- |
| Clinical case discussions | 650 | 54.9 | 52.1 | 57.8 |
| Service/healthcare team meetings | 569 | 48.1 | 45.3 | 50.9 |
| Training, updating and continuing education | 470 | 39.7 | 37.0 | 42.5 |
| Patients’ consultation and guidance | 362 | 30.6 | 28.0 | 33.3 |
| Prescriptions, certificates or medical reports | 292 | 24.7 | 22.3 | 27.2 |
| Patients electronic record annotations | 272 | 23.0 | 20.7 | 25.5 |

**Table A4:** **Types of service provided by physicians as users of telemedicine**

| **SERVICES** | **n** | **CI (95)** | | |
| --- | --- | --- | --- | --- |
| Hospitals (public and private) | 926 | 78.3 | 75.9 | 80.6 |
| Individual practices and private clinics | 786 | 66.4 | 63.7 | 69.1 |
| Public primary care and outpatient care | 691 | 58.4 | 55.6 | 61.2 |
| Administrative non-care services | 462 | 39.1 | 36.3 | 41.9 |
